# Reply to Maleszka et al.: DNA methylation signals persist across insect metamorphosis

**DOI:** 10.1073/pnas.2524479122

**Published:** 2025-10-10

**Authors:** Erin E. B. Foley, Christian L. Thomas, Charalambos P. Kyriacou, Eamonn B. Mallon

**Affiliations:** ^a^Division of Genetics and Genome Biology, School of Biological and Biomedical Sciences, University of Leicester, Leicester LE1 7RH, United Kingdom

Previously (1), we demonstrated that the wasp, *Nasonia vitripennis*, responds to larval diapause with an extended adult lifespan and a slowing of epigenetic aging. We proposed *Nasonia* as a tractable model for dissecting the causal mechanisms of epigenetic aging. Maleszka et al. (2) raise concerns regarding specific analytical procedures and the biological plausibility of DNA methylation signals persisting across insect metamorphosis.

Maleszka et al. state that our model was trained only on control samples and lacked cross-validation. This is not the case. As noted in the *SI Appendix*, we applied 10-fold cross-validation with three repeats. Both diapause and control samples were included in the training set. CpGs were preselected in two steps: first, loci showing a main effect of age in an interaction model containing all samples; second, loci correlating with age in the control group. Preselection was necessary given the limited sample size, to address the “curse of dimensionality” in machine learning (3). Restricting the second step to controls ensured that the CpGs retained were genuinely associated with chronological age in the absence of intervention (4). This strategy created a baseline clock against which deviations from normal aging trajectories could be detected.

They also argue that whole-body, low-coverage methylomes are inappropriate for aging studies. They cite their own approach of ultradeep sequencing at up to 340,000× coverage. This is far beyond current practice: most human clocks are built on arrays equivalent to 50 to 100× whole-genome coverage (5, 6). Similarly, most mammalian clocks are derived from bulk tissues containing multiple cell types (4). Insects are typically studied as whole bodies due to their small size, and this approach has already yielded important epigenetic aging insights (7). We agree that tissues can age at different rates and anticipate that invertebrate clocks will also move toward tissue-specific resolution, as is occurring in mammalian studies.

They suggest that our evidence does not support the claim that epigenetic aging is both measurable and developmentally modifiable. Our study demonstrated both points: we constructed methylation-based age predictors and showed that diapause significantly slowed epigenetic aging. With respect to mechanism, we agree that further work is needed. Our reference to mechanistic target of rapamycin and insulin signaling derived from gene ontology enrichment of clock CpGs and should be viewed as generating hypotheses for future research rather than claims of cis-regulatory causation.

Finally, Maleszka et al. question how larval DNA methylation patterns could be transferred to adults, given extensive tissue remodeling during holometabolous metamorphosis. We disagree that metamorphosis necessarily erases methylation signals. Pegoraro et al. (8) showed that adult *Nasonia* emerging from diapause differed in methylation profiles from controls, and that altering methylation in mothers led exclusively to diapausing offspring. In honeybees, DNA methylation patterns can be transmitted from fathers to their adult daughters, showing that such marks persist through the entire course of development (9). Together with other evidence that large-scale methylation reprogramming does not occur in insect development (10, 11), these findings indicate that DNA methylation signals can survive developmental transitions, consistent with our conclusion that *Nasonia* provides a tractable system for studying the developmental modulation of epigenetic aging.
